# A CPW fed quad-port MIMO DRA for sub-6 GHz 5G applications

**DOI:** 10.1371/journal.pone.0268867

**Published:** 2022-06-10

**Authors:** Assad Iqbal, Jamal Nasir, Muhammad Bilal Qureshi, Aftab Ahmad Khan, Jalil Ur Rehman, Owais, Hamood Ur Rahman, Muhammad A. B. Fayyaz, Raheel Nawaz

**Affiliations:** 1 Department of Electrical and Computer Engineering, COMSATS University Islamabad, Abbottabad, Pakistan; 2 James Watt School of Engineering, University of Glasgow, Glasgow, United Kingdom; 3 Research Directorate, National University of Sciences and Technology (NUST), Islamabad, Pakistan; 4 OTEHM, Manchester Metropolitan University, Manchester, United Kingdom; Edinburgh Napier University, UNITED KINGDOM

## Abstract

The present work investigates a novel four-port, multiple-input multiple-output (MIMO), single element dielectric resonator antenna (DRA) for sub-6 GHz band. The DRA is designed and fabricated into a symmetric cross shape and fed using a coplanar waveguide (CPW) feed. A single radiator with four ports is rarely found in the literature. The -10 dB impedance bandwidth covered by the antenna is from 5.52 GHz to 6.2 GHz (11.6%) which covers fifth generation (5G) new radio (NR) bands N47 and wireless local area network (WLAN) IEEE 802.11a band. The isolation between orthogonal ports is about 15 dB while the isolation between opposite ports is 12 dB. The radiation pattern of the proposed antenna is bidirectional due to the absence of a ground plane below the DRA. The orthogonal modes excited in the DRA are ${TE}_{\delta 21}^{x}$ and ${TE}_{2\delta 1}^{y}$ through the four symmetrical CPW feeds. The simulated and measured results of the proposed design show that MIMO characteristics are achieved by pattern diversity between the ports. Due to the perfect symmetry of the design, the proposed work could be extended to MIMO array applications as well.

## Introduction

The present fourth generation (4G) mobile standard requires the demand for a high data rates and link reliability [1]. Such features can be achieved using multiple transmit and receive antennas at both ends without using extra spectrum and power [1, 2]. However, the present long-term evolution (LTE) and 4G no longer keep up with the ever-increasing demand for low latency and high spectral efficiency. The fifth-generation new radio (5G NR) can deliver 100 times faster data rates as compared to 4G-LTE with a latency of less than 1 ms [3]. Many research projects are also initiated towards the sixth generation (6G) [4–6]. At present, to meet the existing 5G demand new design approaches and novel concepts for antenna design are necessary. Some research works have recently been performed on designing antenna arrays for 5G networks using traditional antenna structures [7–9]. However, due to the size constraint of today’s wireless devices, it is challenging to design multiple-input multiple-output (MIMO) antennas with small size and low mutual coupling [8–10]. In recent years Dielectric Resonator Antenna (DRA) has gained predominant importance in MIMO systems because of its small size, high gain, radiation efficiency, ease of excitation and fabrication [11, 12]. Moreover, multiple modes can be excited in a single radiating element of the DRA. These features make it a suitable candidate for MIMO operation. A dielectric resonator can excite multiple modes in a single DR element. Each mode can carry an individual data stream at each band. For MIMO operation two modes must be excited at the same frequency band, called degenerate mode. If DRA is excited at multiple degenerated modes, distinct data streams can be transmitted simultaneously from each mode at different frequency bands [13]. However, it is challenging to excite multiple modes in a single radiating element for MIMO operation due to mutual coupling between modes.

DRA elements can be excited with multiple feeding ports [14]. A six-port six-element MIMO DRA was proposed in [15], however, such an arrangement makes the MIMO antenna system occupy a larger footprint. The authors in [16] proposed an omnidirectional cylindrical dielectric resonator antenna with dual polarization covering a single band, whereas a similar dual-port, aperture coupled using a single DRA was proposed for worldwide interoperability for microwave access (WiMAX) applications in [17]. A dual-band, eight-port, eight-element MIMO DRA for wireless local area network (WLAN) application was proposed in [18] which covers two bands from 2.38–2.5 GHz and 5.7–6 GHz. The volume of the proposed design is large and bulky because the proposed design has eight resonating elements. A triple port, single element MIMO DRA was proposed for X band application [19] and an L-shape, dual-port, dual-band MIMO DRA was proposed in [20] which covered both WiMAX and WLAN bands. A two-element DRA placed back-to-back with four ports was proposed in [21]. The proposed design is used for a sub-6 GHz band having a bi-directional diversity pattern. All these works have good addition to the literature, but they can be extended for multiple ports to achieve enhanced MIMO performance. In this article, we propose a novel plus shape, four-port, single element, MIMO DRA which covers the WLAN band from 5.52 GHz to 6.2 GHz. To reduce the mutual coupling between all four ports, four symmetrical coplanar waveguides (CPW) feeds are used to excite the orthogonal modes (${TE}_{\delta 21}^{x}$ and ${TE}_{2\delta 1}^{y}$). The advantage of the proposed design is its simplicity, and scalability and can be used for MIMO array applications as well. To the extent of our knowledge, it is the first design of a four-port MIMO DRA with a single resonator having bi-directional pattern diversity for sub 6 GHz 5G band. A single radiator with four ports is rarely found in the literature due to high mutual coupling between ports with a single radiating element which is challenging to be tackled. To resolve this issue, the proposed DRA is fed by four ports. For orthogonal ports (Port 1 and Port 2) the excitation of orthogonal modes (${TE}^{x}$ and ${TE}^{y}$) resulted in low mutual coupling. For non-orthogonal ports (port 1 and port 4) the modes excited are the same but DRA is not a rectangular DRA but is cut into a ‘plus’ shape. By doing so, the portion of the DRA where the internal fields were interfering gets cancelled. In the ‘plus’ shaped DRA the fields excited by the non-orthogonal ports are not interfering with each other and hence low coupling has also been achieved between the non-orthogonal ports.

### Antenna design

**Fig 1:**
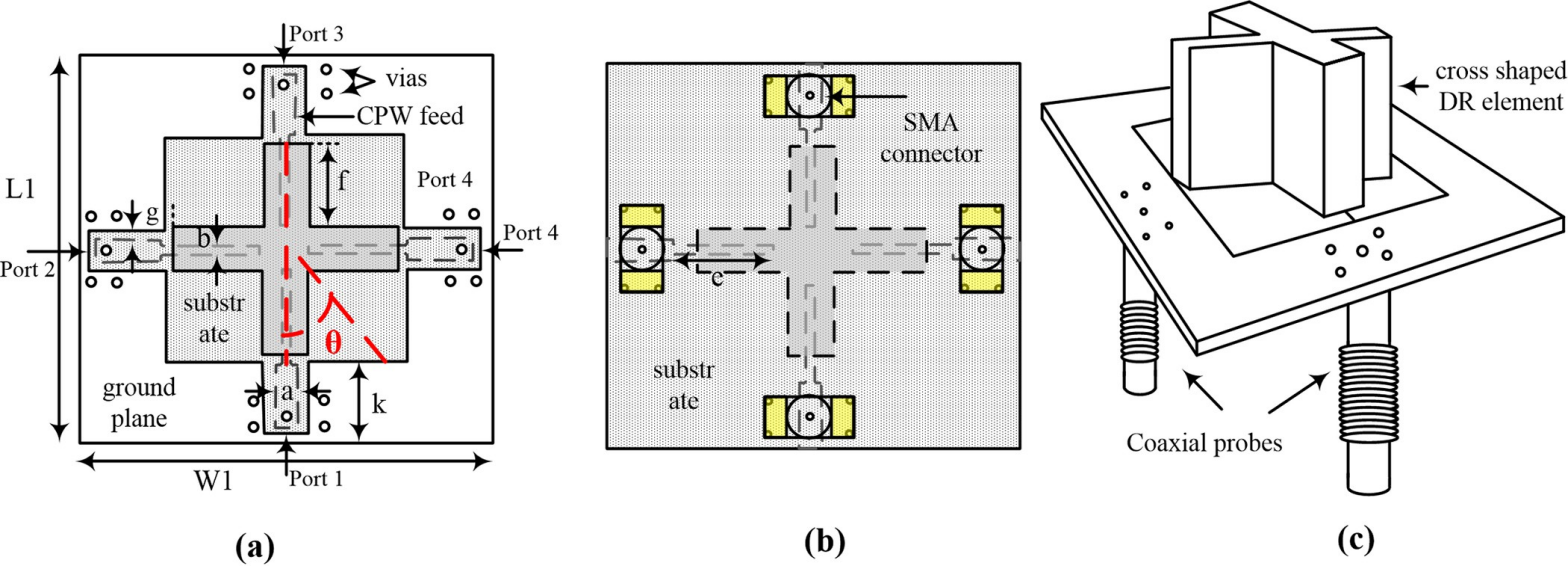
Geometry of the proposed four-port MIMO DRA (a) top view (b) bottom view (c) slanted view of the design. (W1 = L1 = 55 mm, a = 3 mm, b = 1 mm, c = 2.5 mm, e = 11.5 mm, f = 14.6 mm, g = 3.5 mm, k = 9 mm).

The proposed design is composed of a plus-shaped DRA of the dimension of 28×5 mm^2^ and a height of 10mm as shown in Fig 1. The DR material is Eccostock® Hik500f having a permittivity of 10 and loss tangent of 0.002. The DR is placed on an FR-4 substrate having a permittivity of 4.4 and dimensions of 55×55×1.6 mm^3^ (2.11λ_g_ × 2.11 λ_g_ × 0.061λ_g_, where λ_g_ is the guided wavelength at 5.52 GHz). A partial ground plane of dimension 55×10 mm^2^ with some modifications for isolation improvement is etched on the top of the substrate. CPW feed lines are etched on top of the substrate. The feed lines have two-step impedance matching, the first step includes a CPW feed line with a width of 3 mm and a length of 6.5 mm having a gap *c* = 2.5 mm between the ground and CPW for 50 Ω impedance matching. The second step is a feed line of length 14.6 mm and width of 1 mm to excite the DR. In previously published works, the DRA is usually placed on a ground plane which results in a unidirectional radiation pattern. In [17] the authors have placed two DRAs back-to-back to achieve a bi-directional radiation pattern. However, in the proposed work the lack of ground plane below the DRA; a bidirectional radiation pattern is easily achieved.

**Fig 2:**
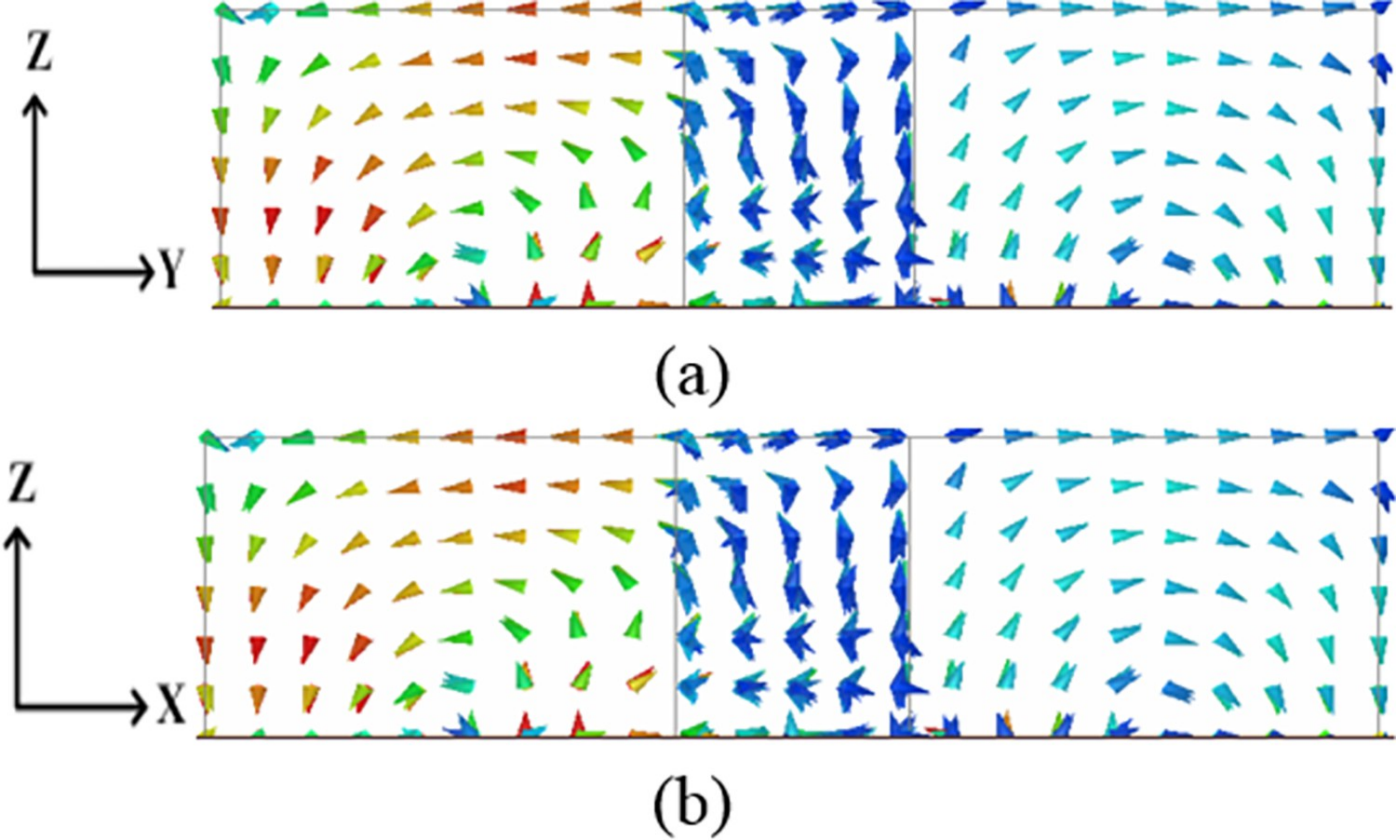
Patterns of electric filed distribution (a) ${TE}_{\delta 21}^{x}$ mode at 5.8 GHz and (b) ${TE}_{2\delta 1}^{y}$ mode at 5.8 GHz.

By using the dielectric waveguide model (DWM) [22], the resonance frequency of a rectangular DR for the ${TE}_{\delta 21}^{x}$ for the said dimensions was calculated to be 5.5 GHz. To make the DRA a four-port MIMO with acceptable bandwidth and isolation, the rectangular DR was converted to a plus-shaped DRA. This changed the resonance frequency to 5.8 GHz. The excited modes inside the plus-shaped DRA are shown in Fig 2. The figures clearly show the excitation of ${TE}_{\delta 21}^{x}$ and ${TE}_{2\delta 1}^{y}$ modes by all four ports.

### Parametric study

The performance of the proposed MIMO antenna depends on various parameters. However, in this research, only those parameters are considered that significantly affect the proposed MIMO antenna performance. Additionally, due to the symmetry of the design, results of reflection coefficient *S*_*11*_, while *S*_*21*_ (orthogonal ports) and *S*_*41*_ (non-orthogonal ports) are shown for isolation.

#### Effect of DR size on isolation and impedance matching

**Fig 3:**
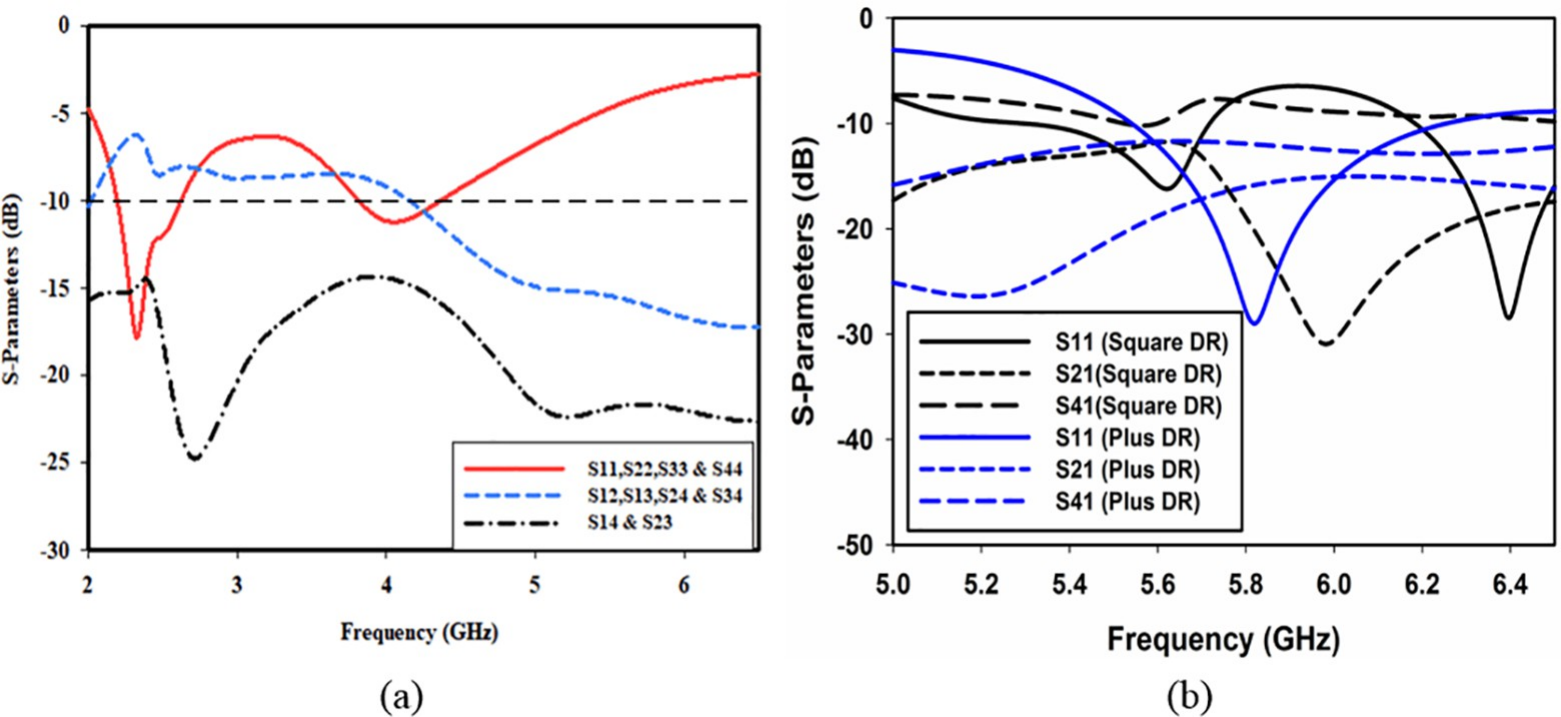
Input reflection (a) without DR and (b) with DR.

The isolation, impedance matching, and resonance frequency of the antenna are dependent on the DR shape and size. Fig 3(A) shows the effect of the DR segment on the performance of the antenna. This figure indicates that without DR, there is no resonance at 5.8 GHz. As the CPW feed line gives resonance at 2.4 GHz and 4 GHz, isolation at these bands is poor as clear from the figure. After placing a square shape DR having dimensions of 28×28×10 mm^3^ on the substrate, the S-parameters reveal that the antenna is resonant at 5.5 GHz, 6.4 GHz and not at the band of interest, as shown in Fig 3(A). Also, the isolation between non-orthogonal ports (*S*_*41*_ long dashed lines in black) is very poor. Once the square shape DR is replaced with plus shape DR, the resonance frequency is adjusted to 5.8 GHz and the isolation between non-orthogonal ports (*S*_*41*_ long dashed lines in blue) improves considerably, as given in Fig 3(B). In both cases, isolation between orthogonal ports (*S*_*21*_ short dashed lines in black and blue) is about 15 dB.

#### Effect of feedline length ‘e’ on impedance matching and isolation

**Fig 4:**
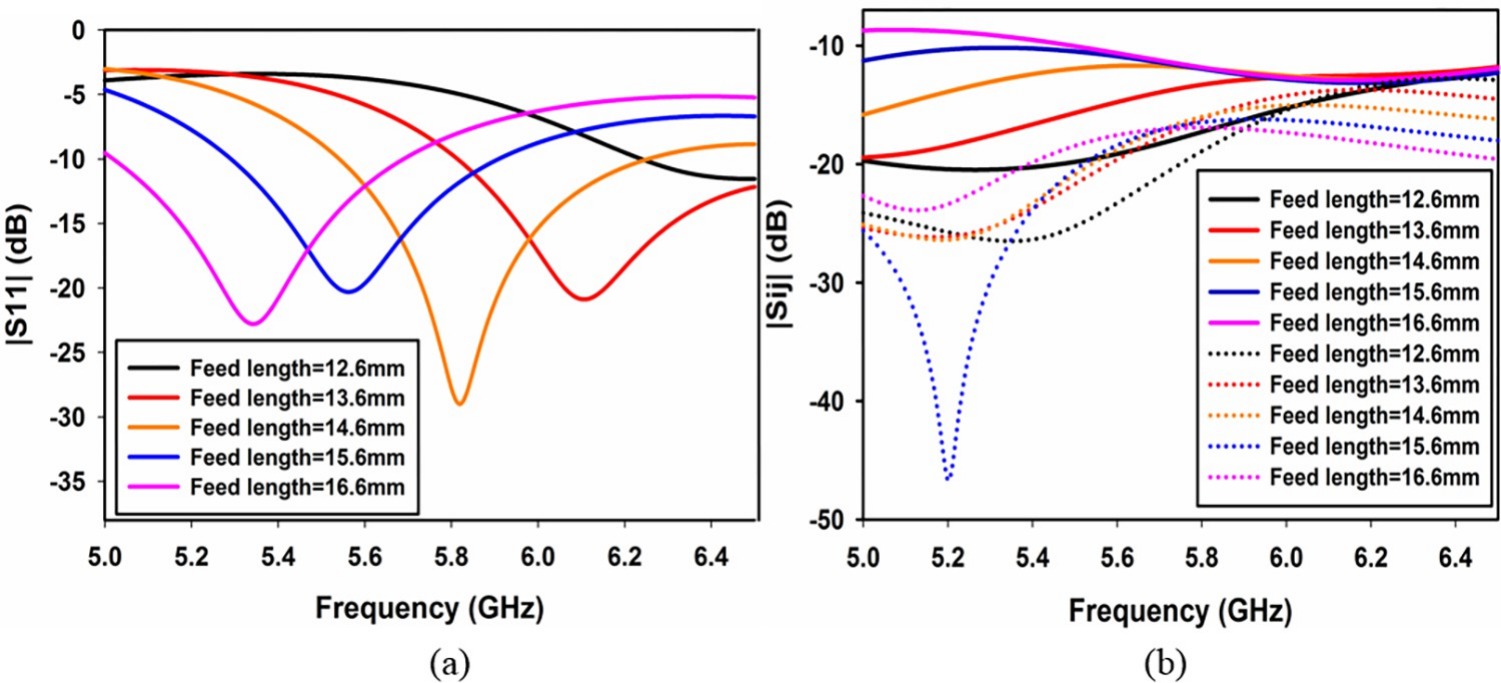
Effect of feedline length ‘*e*’ (a) *S*_*11*_ and (b) *S*_*21*_ & *S*_*41*_.

The feed line length ’*e*’ as shown in Fig 1(B) plays an important role in deciding the resonance frequency and isolation between the ports and is shown in Fig 4(A) and 4(B). Both figures show that by increasing ‘*e*’ the resonance frequency decreases while the isolation between the orthogonal ports (*S*_*21*_ dotted lines) and non-orthogonal ports (*S*_*41*_ solid lines) decreases. The optimum value of ’*e*’ is chosen to be 14.6 mm at which the resonance frequency is 5.8 GHz while *S*_*21*_ and *S*_*41*_ are 16.01 dB and 11.93 dB, respectively.

#### Effect of tilting angle ‘θ’ on the impedance matching and isolation

**Fig 5:**
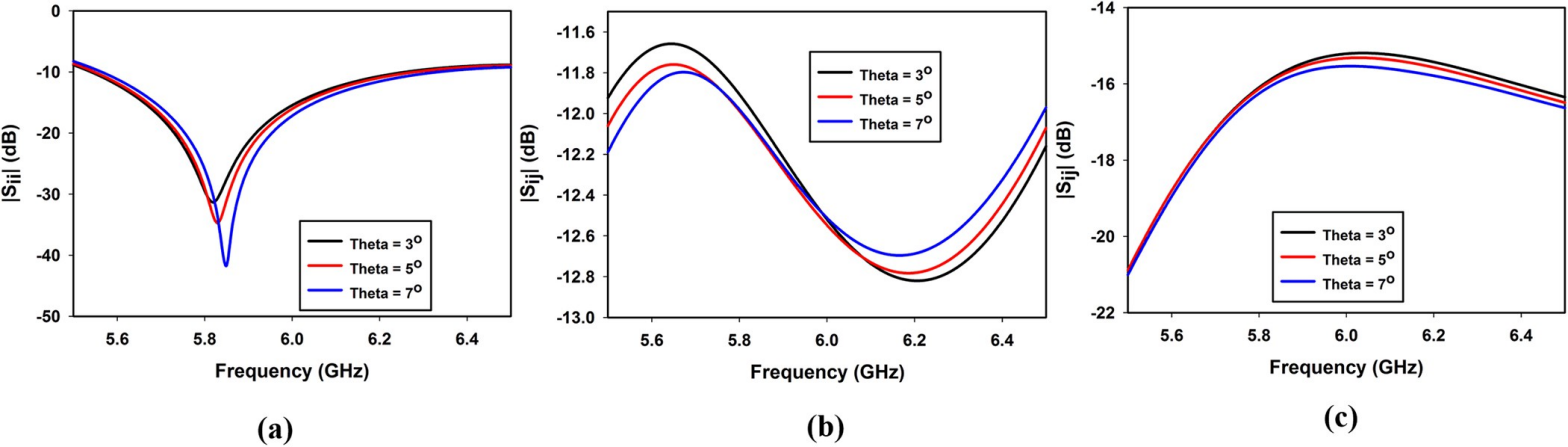
Effect of tilting angle ‘*θ*’ on (a) *S*_*11*_, (b) *S*_*21*_ and (c) *S*_*14*_.

The effect of the tilt angle *θ* (shown in Fig 1(A)) which is the angle between the DR and the x-axis is shown in Fig 5. As clear from Fig 5(A)–5(C), the tilting angle slightly shifts the resonance frequency towards the right while its effect on the mutual coupling between the orthogonal ports (*S*_*21*_) and non-orthogonal ports (*S*_*41*_) is insignificant (Fig 5(B) and 5(C)).

#### Effect of CPW gap ‘g’ on the impedance matching and isolation

**Fig 6:**
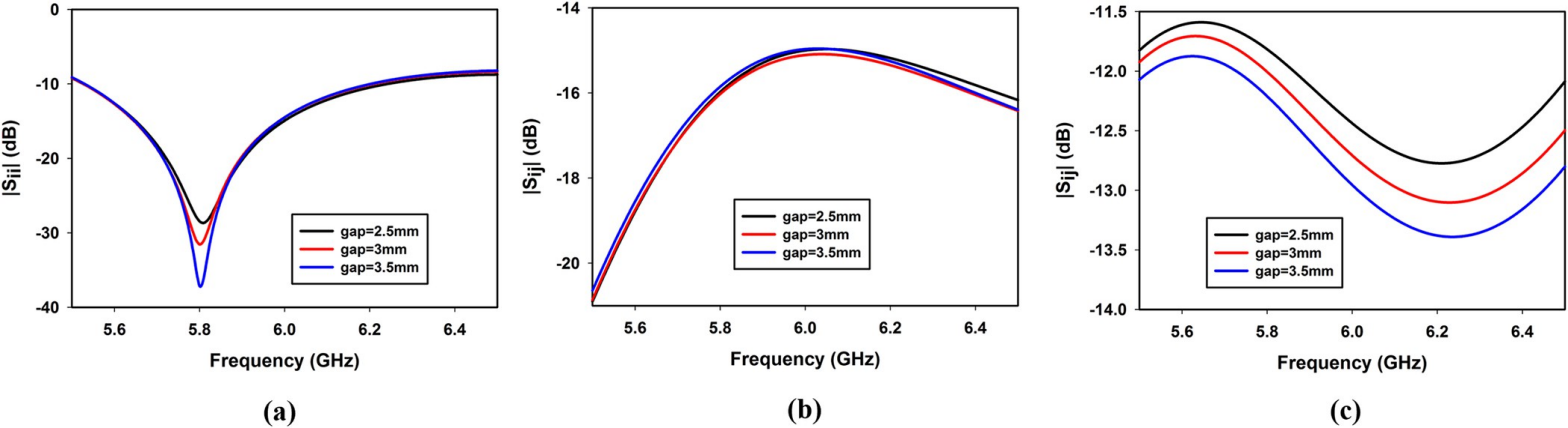
Effect of CPW gap ‘*g*’ on (a) *S*_*11*_, (b) *S*_*21*_ and (c) *S*_*14*_.

The gap ‘*g*’ between the feedline and the ground plane (Fig 1(A)) plays a significant role in the impedance matching and mutual coupling between the ports as shown in Fig 6. Fig 6(A) reveals that by increasing the gap ‘*g*’ matching improves. The coupling between the non-orthogonal ports (*S*_*41*_) also improves by increasing ‘*g*’ as shown in Fig 6(B), while coupling between orthogonal ports (*S*_*21*_) remains almost the same as shown in Fig 6(C). A value of *g* = 3mm has been selected for the proposed design.

## Results and discussions

**Fig 7:**
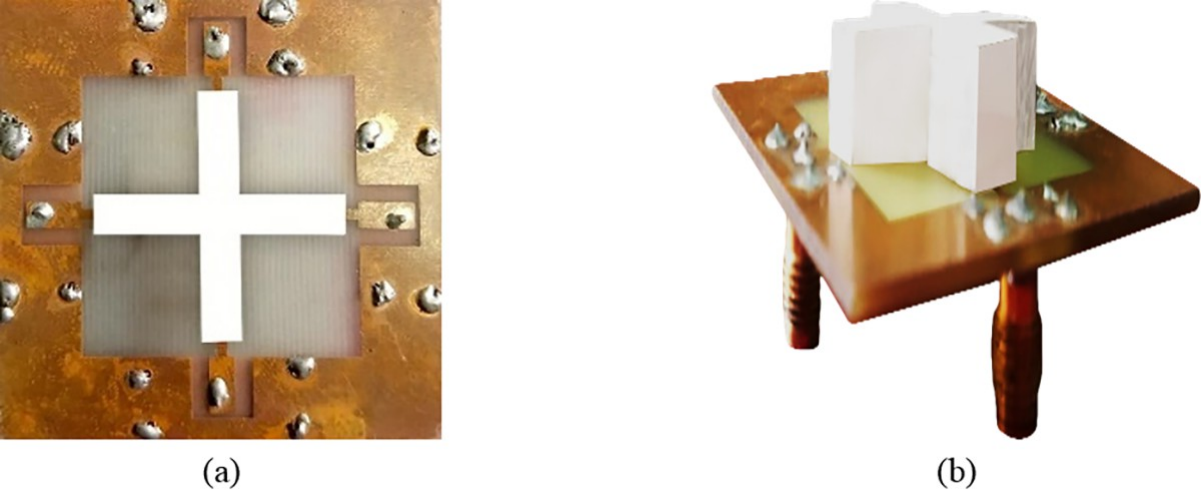
Fabricated DRA (a) top view and (b) perspective view.

Fig 7 shows the prototype of the proposed four-port MIMO DRA antenna. The measurement of the proposed antenna has been carried out by using the E5071C vector network analyzer (VNA).

**Fig 8:**
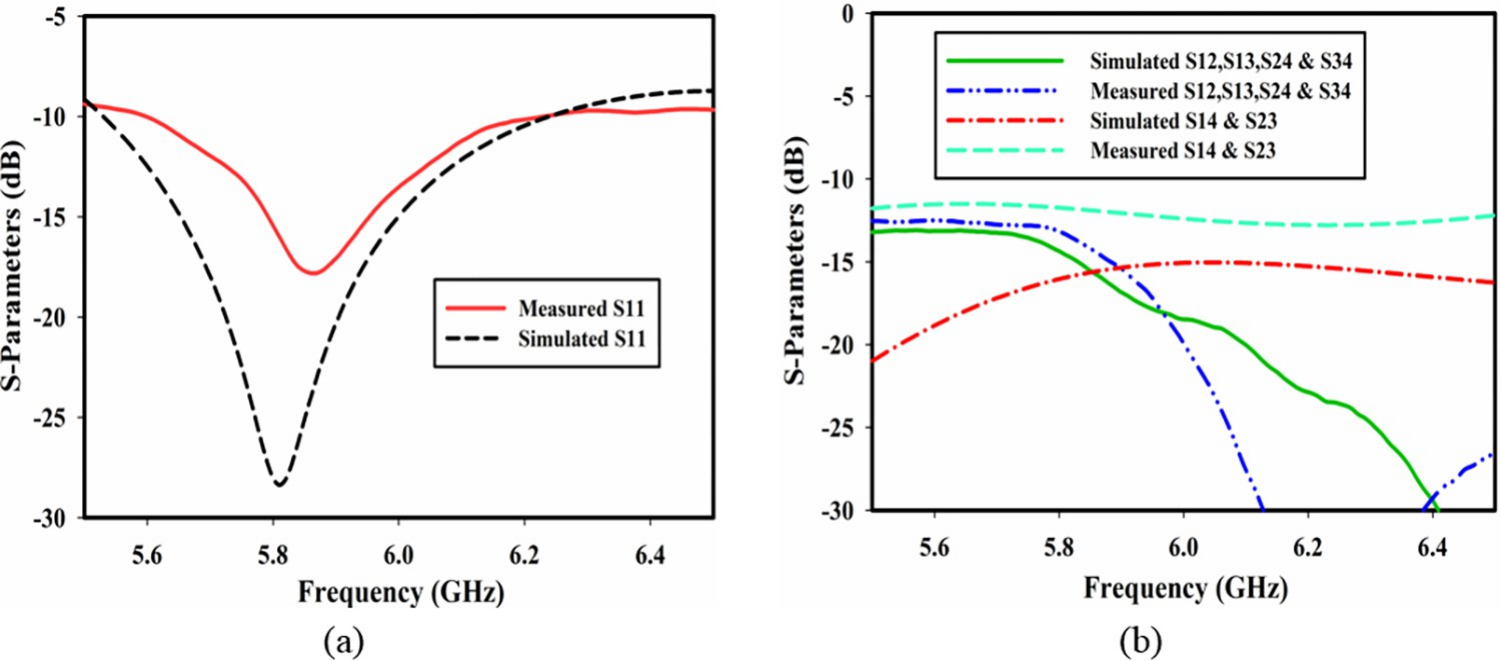
Simulated and measured results of the proposed MIMO DRA (a) reflection coefficient and (b) isolation.

The simulated and measured S-parameters of the proposed MIMO antenna are shown in Fig 8. Fig 8(A) shows the simulated and measured reflection coefficient of the antenna. As clear from the figure, there is a mismatch in simulated and measured results due to fabrication tolerance. However, the matching is still better in the frequency band of interest, i.e., 5.8 GHz. Fig 8(B) shows isolation between different ports. It is seen in the figure that the isolation between orthogonal ports P1-P2, P1-P3, P2-P4 and P3-P4 is well below 15 dB in the band of interest while the isolation between non-orthogonal ports P1-P4 and P2-P3 is up to 12 dB in the band of interest.

**Fig 9:**
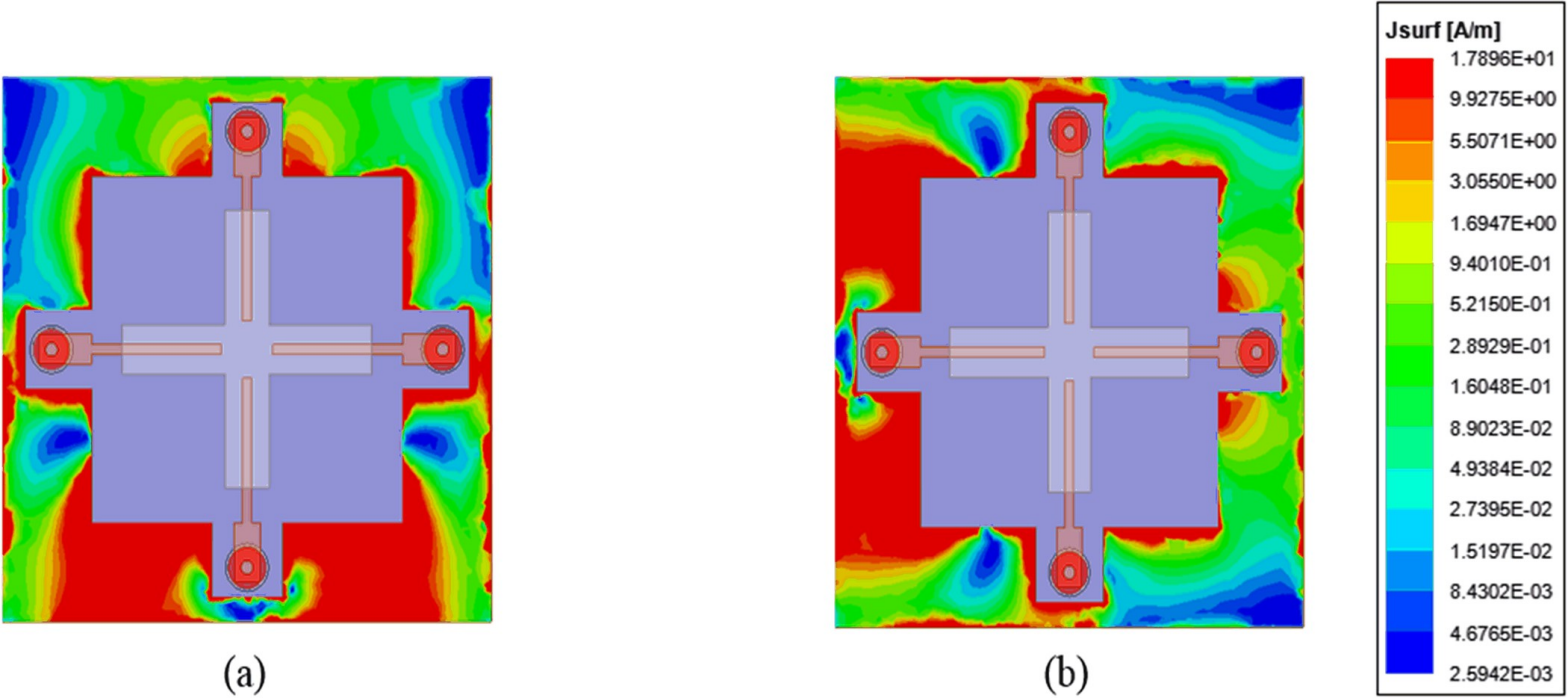
Current distribution (a) port 1 excited and (b) port 2 excited.

The isolation between the ports can be explained with the help of surface current flow on the ground plane and E-field distribution inside the DR. As evident from Fig 9(A), when port 1 is excited and other ports are terminated with 50 Ω, most of the current is confined to the exciting port. With the optimized shape of the ground plane, the current flow towards the rest of the ports is minimal. The same is the case when port 2 is excited as shown in Fig 9(B). However, the cause of coupling between ports is highly dependent upon the excited modes inside the DR.

**Fig 10:**
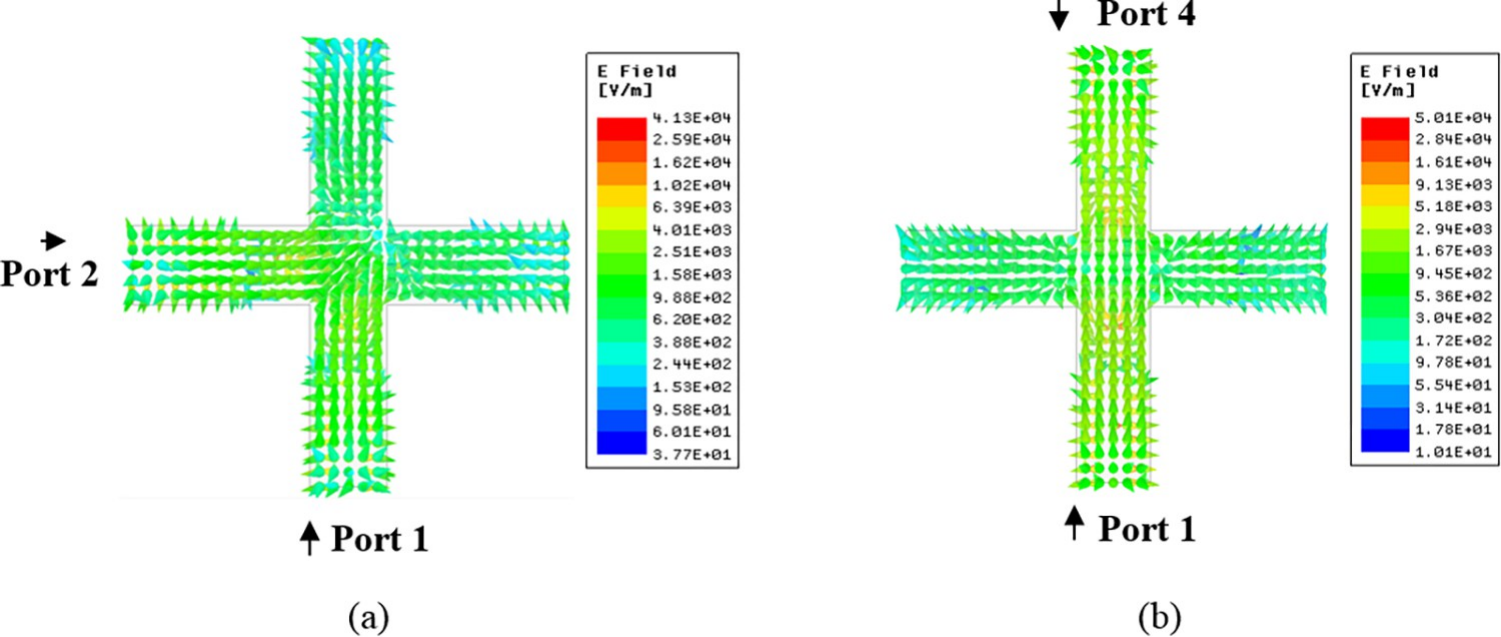
E-field Distribution inside DR (a) Orthogonal ports (P1-P2) and (b) non-orthogonal ports (P1-P4).

The modes excited are orthogonal (${TE}_{\delta 21}^{x}$ and ${TE}_{2\delta 1}^{y}$) when orthogonal ports (P1-P2, P1-P3, P2-P4, P3-P4) are active resulting in low coupling between these ports as shown in Fig 10(A). From this figure, it can be deduced that the fields excited are the orthogonal fields (${TE}_{\delta 21}^{x}$ and ${TE}_{2\delta 1}^{y}$) resulting in low coupling between these ports.

On the other hand, when non-orthogonal ports (P1-P4, P2-P3) are active, they result in the excitation of non-orthogonal modes ${TE}_{\delta 21}^{x}$ or ${TE}_{2\delta 1}^{y}$ resulting in high coupling between these ports because of the same polarization as shown in Fig 8(B).

**Fig 11:**
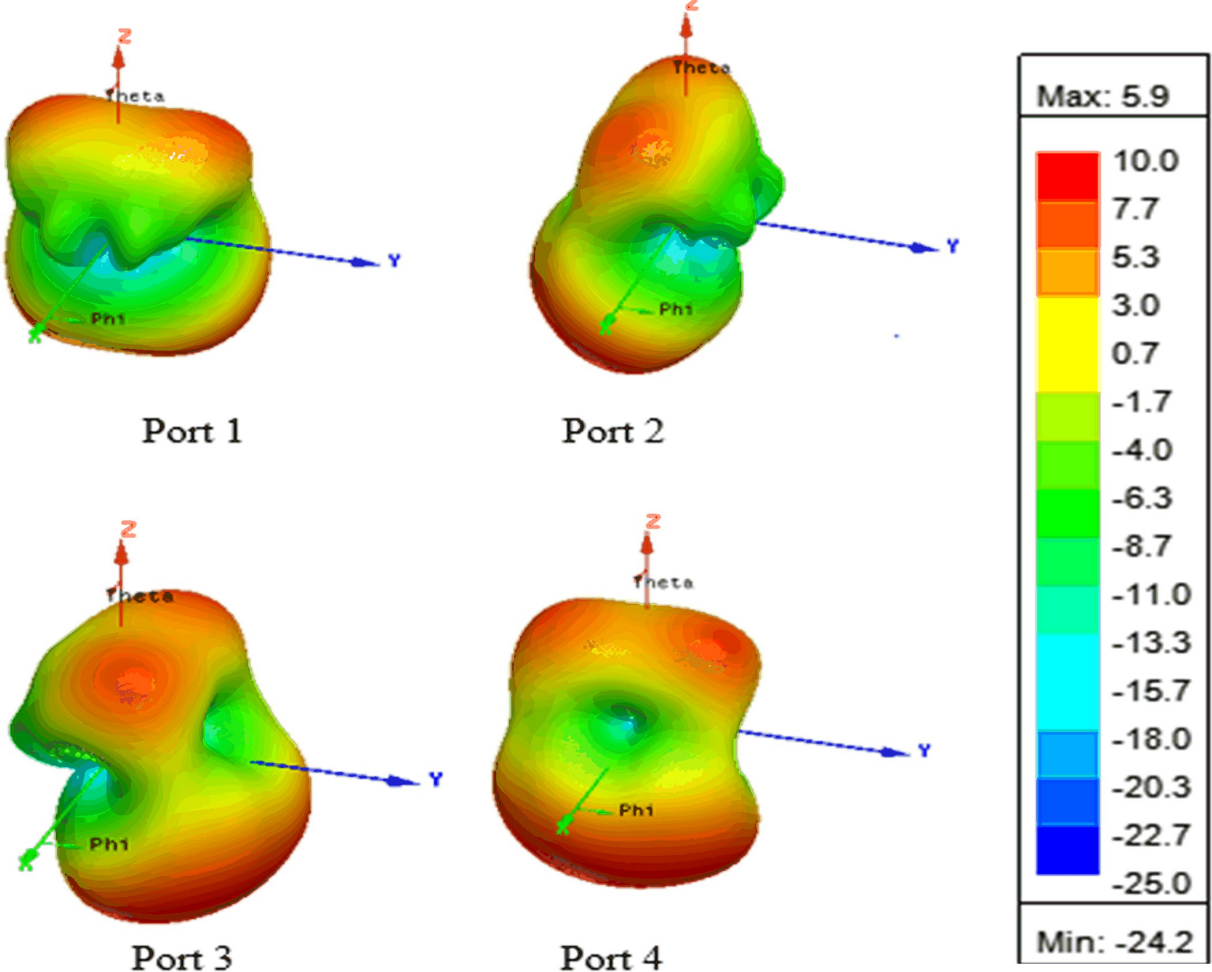
Simulated 3D radiation patterns with ports excited at 5.8 GHz.

The simulated 3D radiation pattern of the proposed four-port MIMO DRA is shown in Fig 11. The pattern diversity is significantly visible which is imperative for MIMO operation. The radiation pattern of port 1 and port 4 are anti-parallel while port 1 and port 2 are perpendicular to each other.

**Fig 12:**
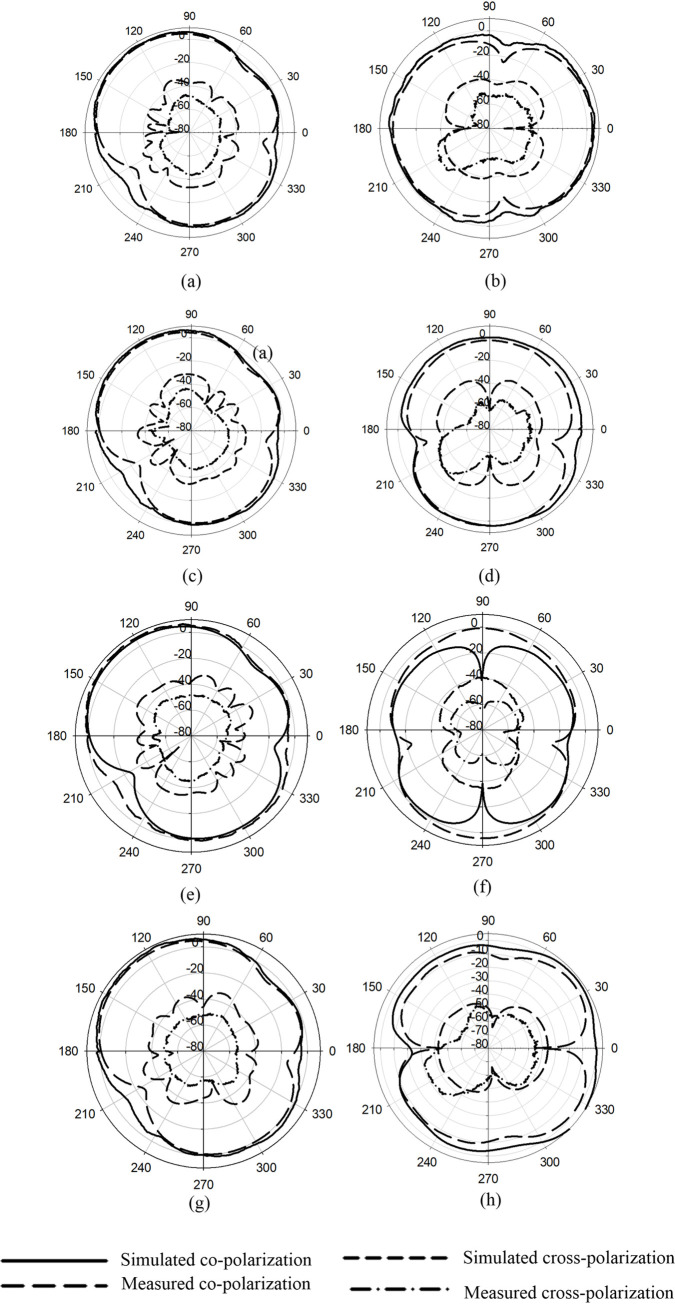
2D gain pattern at 5.8 GHz (a) Port 1 *E-plane* (b) Port 1 *H- Plane* (c) Port 2 *E- Plane* (d) Port 2 *H- Plane* (e) Port 3 *E-plane* (f) Port 3 *H- Plane* (g) Port 4 *E- Plane* and (h) Port 4 *H- Plane*.

The simulated and measured 2D radiation patterns (co and cross-polarization components) of the proposed MIMO antenna are shown in Fig 12. The radiated patterns for all ports in the *H-plane* are pointing in different directions. While, in the *E-plane*, the patterns of P1-P4 and P2-P3 are pointing in the same direction because these ports are non-orthogonal ports. The measured gains of P1, P2, P3, and P4 are 5.2, 5.04, and 4.8.4.3 dBi, respectively. The cross-polarization components in all the cases are less than -30 dB from the Co-polarization component.

The MIMO performance of the proposed MIMO antenna is evaluated in terms of envelope correlation coefficient (*ECC*), diversity gain (*DG*), mean effective gain (*MEG*) and channel capacity loss (*CCL*).

The correlation coefficient (ρ) is a measure of how much communication channels are correlated or isolated from each other. This performance metric takes into account how much the radiation patterns are correlated when operated simultaneously. The envelope correlation coefficient (*ECC*) is the square of ρ, and can be calculated using two methods (Eqs (1) and (2) of [3]), scattering parameters and 3D radiation pattern [23].

In the first method *ECC* only depends upon surface currents. This method is simple us use but do not account for coupling caused by radiation pattern. In the second method, *ECC* also accounts for coupling caused by 3D patterns. This method is more accurate however, it is difficult to use because it requires 3D pattern measurement [24].

$$\rho =\frac{\iint_{4\pi }{\bar{E}}_{1}(\theta ,\varphi ){\bar{E}}_{2}^{*}(\theta ,\varphi )d\Omega }{\sqrt{\iint_{4\pi }{\left|{\bar{E}}_{1}(\theta ,\varphi )\right|}^{2}d\Omega \iint_{4\pi }{\left|{\bar{E}}_{2}(\theta ,\varphi )\right|}^{2}d\Omega }}$$ (1)

$$\rho =\frac{{\left|S_{ii}^{*}S_{ij}+S_{ji}^{*}S_{jj}\right|}^{2}}{\left(1-({\left|S_{ii}\right|}^{2}+{\left|S_{ji}\right|}^{2}))+(1-({\left|S_{jj}\right|}^{2}+{\left|S_{ij}\right|}^{2})\right)}$$ (2)

where ${\bar{E}}_{i}(\theta ,\varphi )$ is the three-dimensional field radiation pattern of the antenna when the ith port is excited, and Ω is the solid angle. (*) is the Hermitian product operator.

**Fig 13:**
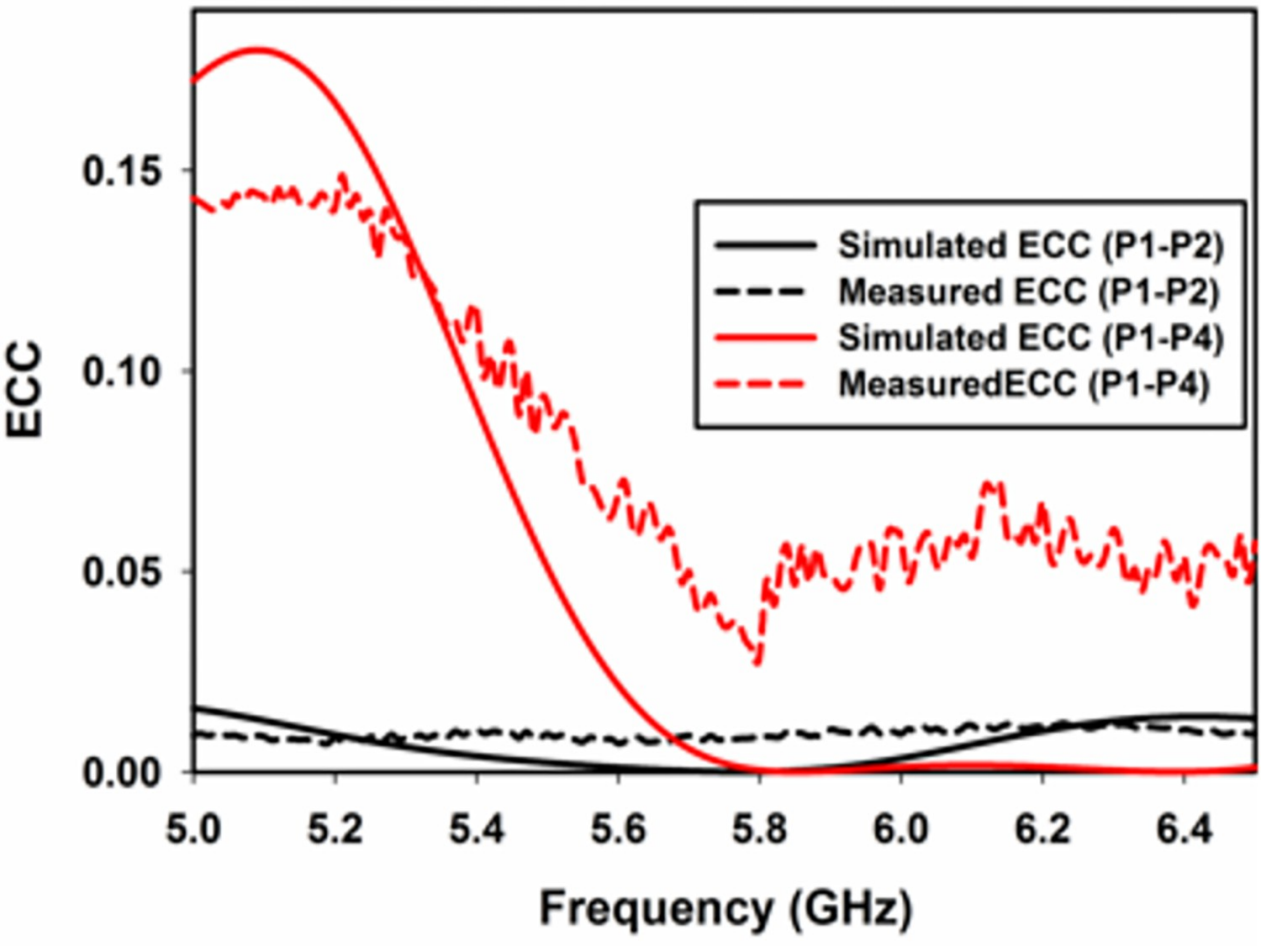
Simulated and measured *ECC*.

The results of *ECC* by using Eq (2) are shown in Fig 13. It can be observed that in the band of interest *ECC* for orthogonal (P1-P2) is below 0.02 while for non-orthogonal ports (P1-P4) the *ECC* is below 0.05. The *ECC* value obtained by using Eq (1) is 0.042 for orthogonal ports (P1-P2) and 0.06 for non-orthogonal ports (P1-P4). The above values clearly show that the *ECC* for the orthogonal ports (P1-P2) is better than the non-orthogonal ports (P1-P4) by using both Eq (1) and Eq (2), as expected.

**Fig 14:**
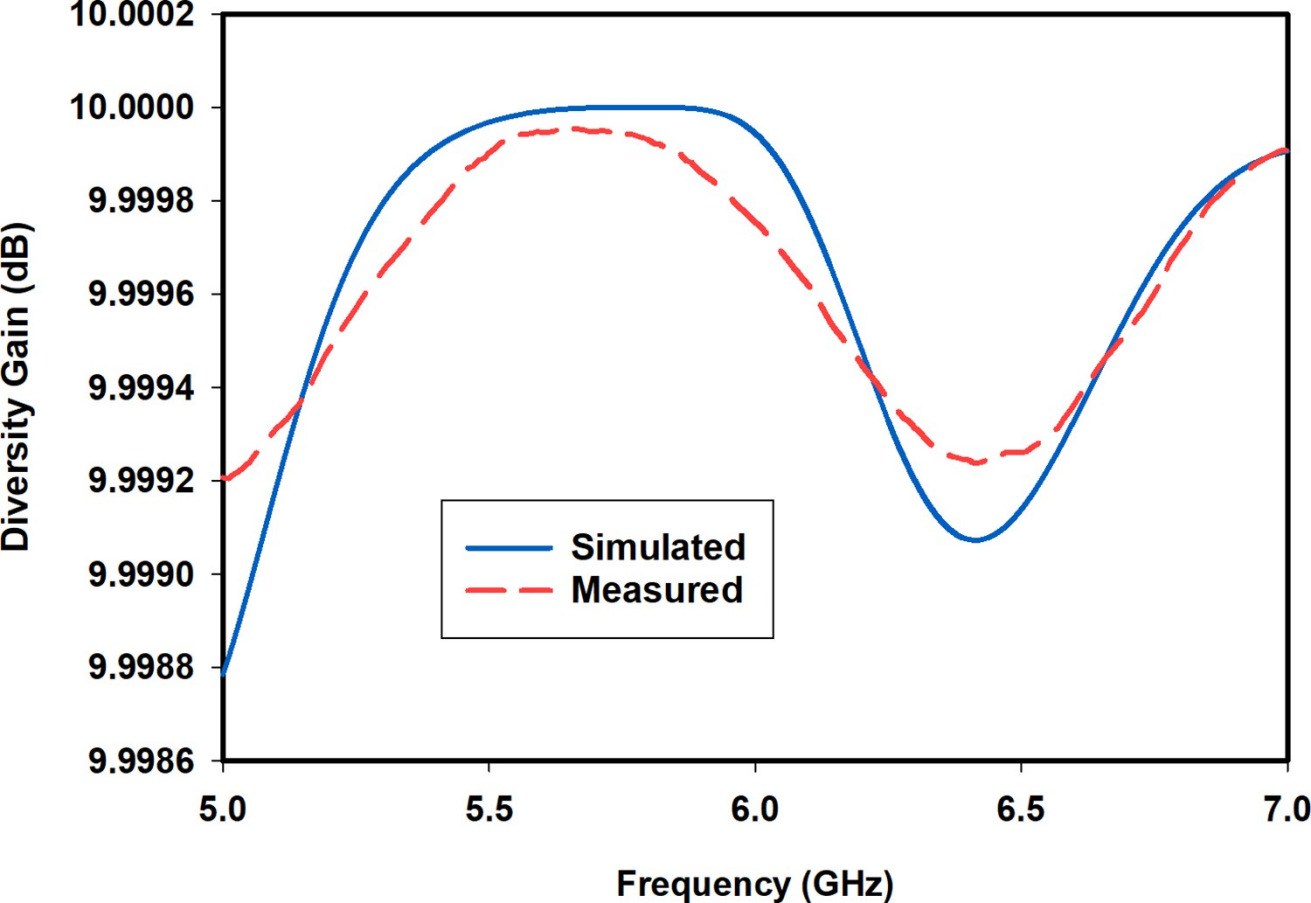
Simulated and measured *DG*.

The diversity gain (*DG*) specifies the quality and reliability of a wireless communication link [25]. A high value of *DG* close to 10 dB is desirable in the operating band. The *DG* is calculated from the *ECC* by using Eq (3). The simulated and measured *DG* of the proposed antenna is shown in Fig 14. It is clear from the figure that the *DG* is close to 10 dB in the band of interest.


$$DG=\sqrt{1-{\left|\rho \right|}^{2}}$$ (3)

**Fig 15:**
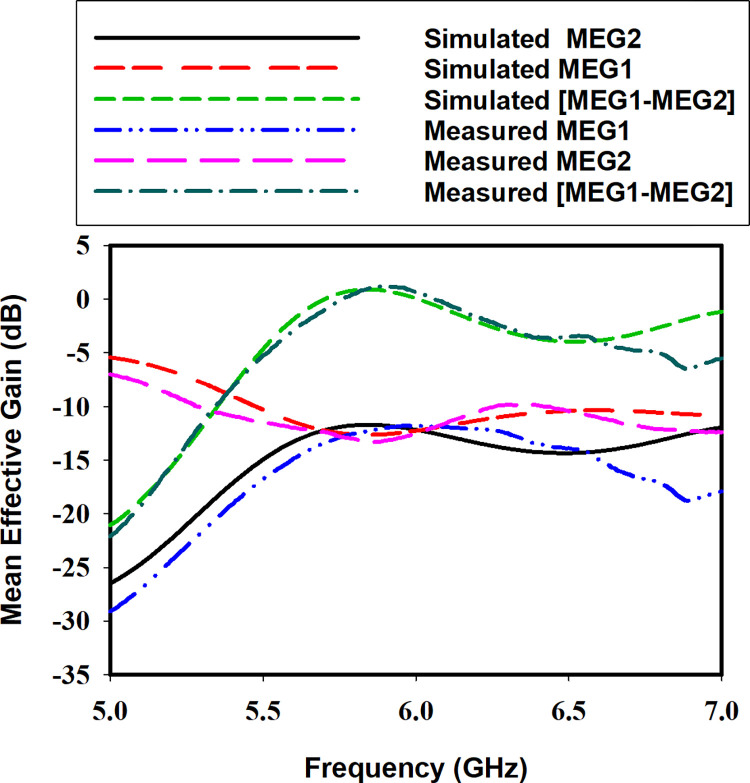
Simulated and measured *MEG*.

Mean effective gain (*MEG*) is another important diversity parameter. It gives the ratio between the diversity antenna received power to the isotropic antenna received power. The ratio of the *MEGs; MEG-1/MEG-2*, *MEG-1/MEG-3*, *and MEG-2/MEG-4* between the ports of the MIMO antenna should be less than 3 dB [25]. The *MEG* is calculated from the S-parameters by using Eqs (4) and (5). For the proposed antenna the simulated and measured *MEG* is presented in Fig 15. As clear from the figure the ratio of the *MEGs* is below 3 dB resulting in acceptable diversity performance.


$$MEGi=0.5[1-{\left|S_{ii}\right|}^{2}-{\left|S_{ij}\right|}^{2}]$$ (4)

$$MEGj=0.5[1-{\left|S_{ij}\right|}^{2}-{\left|S_{jj}\right|}^{2}]$$ (5)

**Fig 16:**
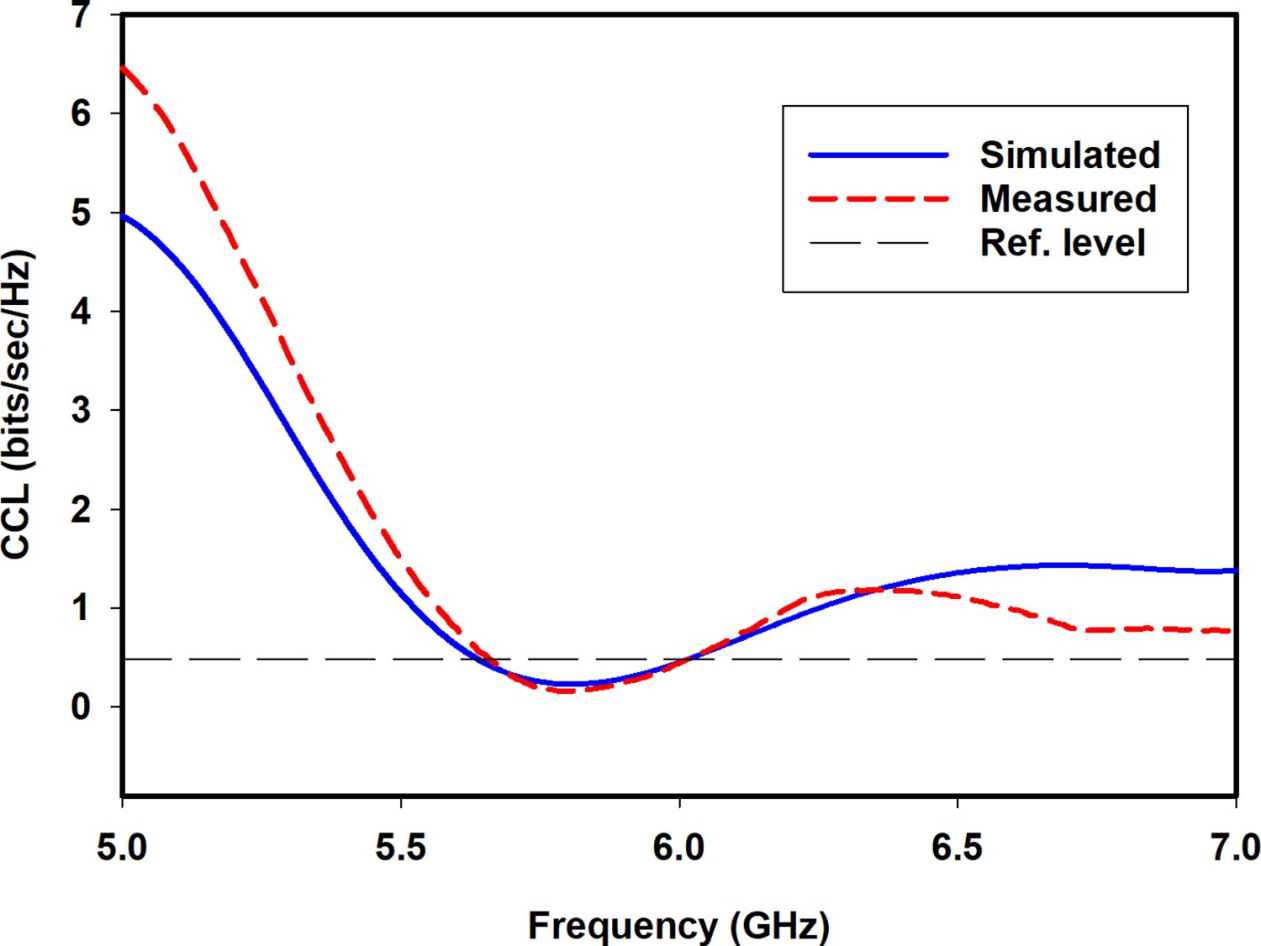
Simulated and measured *CCL*.

The channel capacity loss (*CCL*) approximates the transmission of the maximum limit of the message signal without communication channel loss. The acceptable value of *CCL* should be less than 0.4 bits/s/Hz [25]. The *CCL* is calculated by using S-parameters as in Eq (6). Fig 16 shows the simulated and measured *CCL* of the proposed antenna. As clear from the figure, the *CCL* in the band of interest is below 0.4 bits/s/Hz.

$$CCL=-\log_{2}|A^{R}|$$ (6A)

$$A^{R}=[\begin{array}{llll} A_{11} & A_{12} & A_{13} & A_{14}\\ A_{21} & A_{22} & A_{23} & A_{24}\\ A_{31} & A_{32} & A_{33} & A_{34}\\ A_{41} & A_{42} & A_{43} & A_{44} \end{array}]$$ (6B)

where

$$A_{ii}=1-|\sum_{n=1}^{N}{S_{in}}^{*}S_{ni}|\;for\;i,\;j=1,\;2,\;3\;and\;4$$ (6C)

$$A_{ij}=1-|\sum_{n=1}^{N}{S_{in}}^{*}S_{nj}|\;for\;i,\;j=1,\;2,\;3\;and\;4$$ (6D)

A comparison of the proposed work with previous work is given in Table 1. The proposed MIMO antenna compared to other MIMO antennas available has a single DR element with four ports thus considerably decreasing the size of the MIMO system with reasonable antenna and MIMO performance.

**Table 1:** Comparison of the proposed MIMO DRA antenna with other published MIMO DRA antenna.

Reference	Size (mm^3^)	No. of elements	No. of ports	Isolation (dB)	ECC	Maximum Gain (dBi)	Covered Bands (GHz)
[19]	56.6× 56.6×13.3	1	3	20 dB	0.002	7.5	9.12–9.14
[20]	50×50×7	1	2	16	0.002	5.2	3.4–3.7and 5.15–5.35
[21]	30×30×13.6	1	4	18	< 0.25	5	5.4–6.0
[26]	150×150×14.8	8	8	17	0.122	6.5	2.38–2.5and 5.7–6
[27]	50×50×15	1	2	25	<0.002	5.3	2.51–2.83
[28]	100×100×20	1	2	20	0.02	6.9	1.63–1.84 2.43–2.71 3.28–3.73
[29]	60×60×21.8	1	2	>18	0.15	6	7.29 to 10.65
[30]	80×80×10	4	4	16	0.0045	6.5	3.63–4.25
[31]	37.6×37.6×5	2	2	15.6	0.0059	5.36	3.5–10.9
This work	55×55×10	1	4	15	0.05	5.12	5.52–6.2

## Conclusion

This paper presents the design, implementation, and measurement of a CPW fed, quad-port MIMO DRA with a single resonator for 5G N47 and WLAN applications. The proposed design is a symmetrical plus-shaped DR and excited through four independent CPW feed lines with a common ground plane. The proposed design is bi-directional due to the lack of a ground plane at the bottom. The isolation was achieved by the excitation of orthogonal modes and a modified ground plane. The proposed design has measured gains for P1, P2, P3, P4 of 5.2, 5.04, and 4.8.4.3 dBi, respectively, and radiation efficiency up to 94%. Measured results including antenna performance parameters like input reflection, isolation and radiation patterns are shown which bear close agreement with simulated results. MIMO performance parameter like *ECC*, *DG*, *MEG* and *CCL* shows that the proposed design is an appropriate candidate for MIMO applications at the WLAN band.
